# Association between varicose veins and occurrence of dementia: A nationwide population-based cohort study

**DOI:** 10.1371/journal.pone.0322892

**Published:** 2025-04-30

**Authors:** Ho Geol Woo, Ju-young Park, Moo-Seok Park, Tae-Jin Song

**Affiliations:** 1 Department of Neurology, Kyung Hee University College of Medicine, Seoul, Republic of Korea; 2 Department of Statistics, Yeungnam University, Gyeongsan, Gyeongbuk, Republic of Korea; 3 Department of Neurology, Seoul Hospital, Ewha Womans University College of Medicine, Seoul, Republic of Korea; All India Institute of Medical Sciences - Patna, INDIA

## Abstract

While varicose vein (VV) and dementia are frequent health problems, research investigating association between these conditions has been limited. We aimed to investigate the relationship between the presence of VV and the development of dementia, as well as to evaluate whether treatment for VV correlates with the occurrence of dementia in a longitudinal study involving the general population. Our study included 430,875 participants based on health screening results conducted from 2005 to 2010 in the South Korean health screening cohort database. Presence of VV was defined with at least two or more claims based on International Classification of Diseases, Tenth Revision (ICD-10) of I830-832, I839, or I868. Propensity score matching at a ratio of 1:5 was employed to categorize the participants into two groups based on the presence and treatment of VV, respectively. Primary outcome was the incidence of all-cause dementia with two or more claims based on ICD-10 code (F00-03, G30, and G31), and secondary outcomes considered occurrence of Alzheimer’s disease (AD; F00 or G30) and vascular dementia (VD; F01). Among included participants, presence of VV were noted in 5,096 (1.3%) participants. During a median follow-up of 13.33 (interquartile range 10.4–16.26) years, 55,329 (13.9%) cases of all-cause dementia have occurred. In multivariable analysis, VV group showed increased risk of all-cause dementia compared to non-VV group (hazard ratio [HR]: 1.235, 95% confidence interval [CI]: 1.147–1.329). Contrary to AD, treatment of VV group was significantly associated with decreased risk of VD (HR: 0.566, 95% CI: 0.382–0.841). Our study showed that presence of VV may be associated with an increased risk of future all-cause dementia, and treatment of VV was likely to reduce the incidence risk of VD.

## Introduction

Varicose veins (VV) are a prevalent medical condition affecting populations worldwide [1,2]. Currently, it is reported that the global prevalence of VV ranges from 2% to 73% of the population, with estimates ranging from 2% to 73% in women and 2% to 56% in men, depending on geographic location and study methodologies [1,3]. Characterized by enlarged and twisted superficial veins with a diameter of three millimeters or more, they primarily impact the saphenous veins, their branches, or other superficial veins in the legs [4,5]. Several factors contribute to the development and progression of VV, including female sex, multiple pregnancies, obesity, chronic constipation, and venous thrombosis [6]. More recently, aging has been recognized as an independent risk factor for VV [7,8]. Age-related vascular changes have been linked not only to VV but also to vascular cognitive impairment and neurodegenerative diseases [9].

Previous studies showed that VV, along with venous hypertension and valvular reflux, has been associated with organ damage, including white matter changes in the brain [5,6,10]. Some studies suggest that VV and chronic venous diseases are associated with increased inflammation and endothelial dysfunction, potentially triggering neuroinflammatory pathways linked to dementia and vascular cognitive impairment. Also, these conditions are increasingly recognized as contributors to cerebrovascular damage and cognitive decline [5,11,12]. Additionally, venous pathology may impair cerebral venous drainage, worsening cerebral small vessel disease and white matter lesions—both known risk factors for cognitive decline and dementia [13]. Given these potential pathophysiological mechanisms, further investigation into the association between VV and dementia could provide new insights for dementia prevention and management.

Dementia encompasses a range of disorders marked by persistent and progressive memory loss, cognitive decline, and behavioral changes that disrupt daily functioning [14]. Approximately 90% of dementia cases display neuropathological characteristics consistent with Alzheimer’s disease (AD) or vascular dementia (VD) [15]. With the global population aging, dementia incidence is projected to notably increase. According to published data on the average age of dementia onset in older adults, dementia typically develops around the ages of 83–87 [16,17]. In a study on the age-specific incidence rates of dementia, the incidence rates per 1,000 person-years were reported as follows: for males, 66 years at 8.3, 80 years at 33.7, and 90 years at 87.7; for females, 66 years at 7.2, 80 years at 35.6, and 90 years at 95.7 [18]. Another study found that the percentage of adults with a dementia diagnosis increased with age, from 1.7% in those aged 65–74 to 13.1% in those aged 85 and older [19]. In the United States, approximately 10% of individuals aged 65 and older have dementia, with this prevalence rising sharply in advanced age groups. Specifically, about 3% of adults aged 70–74 are affected, increasing to 22% among those aged 85–89, and reaching 33% in individuals aged 90 and above [20]. While the exact causes of dementia remain unclear, factors such as genetics, environment, neuroinflammation, and vascular damage are thought to play significant roles in its development [21–23]. Moreover, damage to target organs resulting from venous issues is known to contribute to the onset or exacerbation of white matter changes [10,13]. Given the widespread occurrence of VV and dementia, it is crucial to explore potential links between them. However, longitudinal cohort studies providing data to investigate the association between VV and subsequent dementia incidence are currently limited [24,25].

We hypothesized that presence and treatment of VV would be associated with occurrence of dementia. Our aim was to investigate the relationship between the presence of VV and the development of dementia, as well as to evaluate whether treatment or procedure for VV correlates with the occurrence of dementia in a comprehensive longitudinal study involving the general population.

## Materials and methods

### Data source

This study utilized data from the National Health Insurance Service-Health Screening (NHIS-HEALS) cohort database in South Korea, covering the period from 2002 to 2019. The NHIS-HEALS database comprises a biennial nationwide health screening program that is provided free of charge to all adults aged 40 and above in South Korea. The geographical distribution of this database is visualized as shown in S1 Fig. Previous reports have provided detailed descriptions of the dataset (S1 Appendix) [26–29]. To construct the NHIS-HEALS database, a sample cohort was first selected from the 2002 and 2003 health screening participants, who were aged between 40 and above in 2002 and followed up through 2019. This cohort included 514 866 health screening participants who comprised a 10% simple random sample of all health screening participants in 2002 and 2003. The dataset includes demographic information such as sex, age, and household income, as well as data on participants’ health claims, insurance status, and mortality, with records available up to December 31, 2019.

### Study population

**Fig 1:**
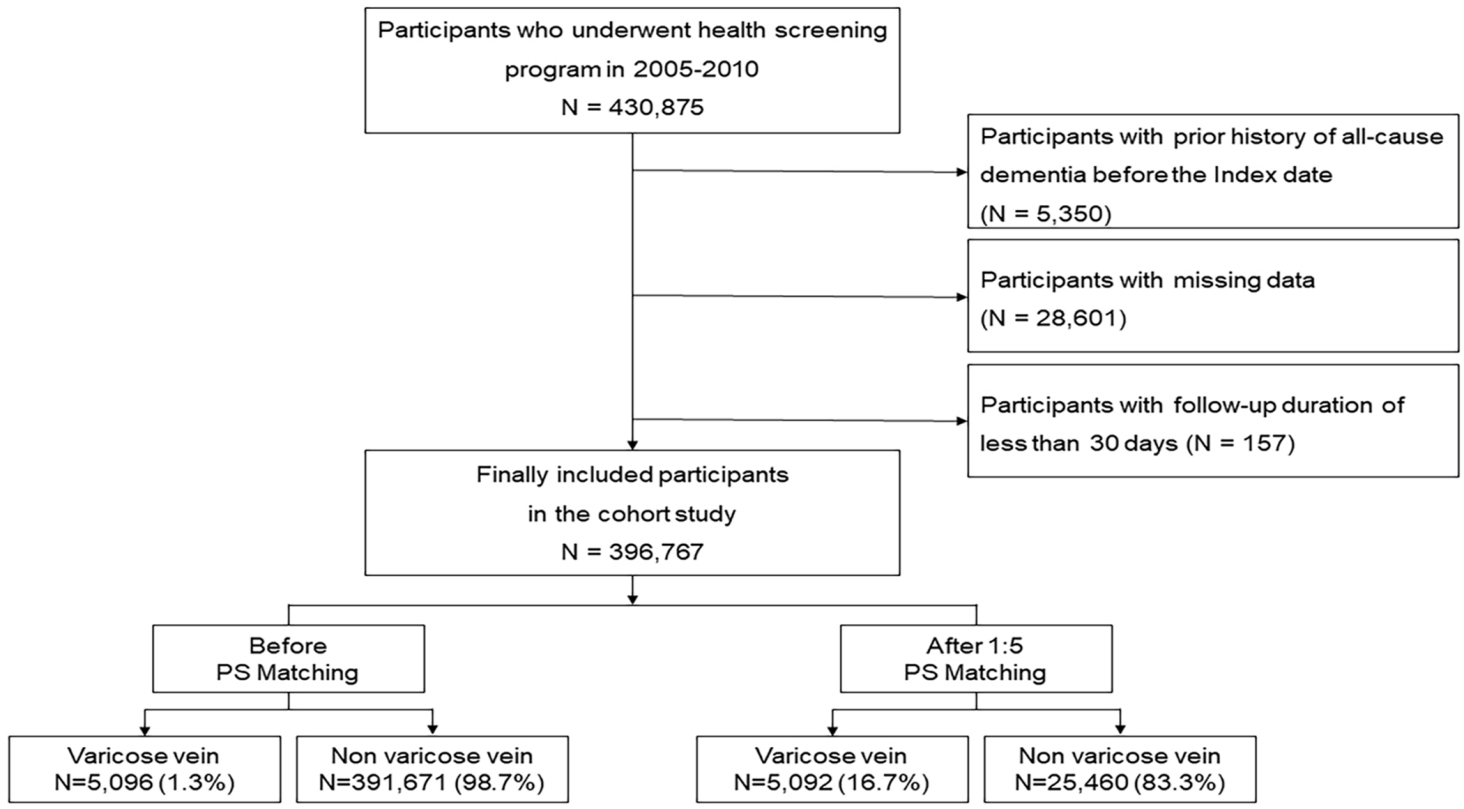
Flow chart of inclusion and exclusion criteria.

This study analyzed 430,875 participants from the NHIS-HEALS database who had undergone the national health screening program between 2005 and 2010. To exclude individuals with a history of all-cause dementia, a washout period was established from January 1, 2002, to the baseline period (2005–2010). During this time, individuals diagnosed with all-cause dementia, as identified using the International Classification of Diseases-10 (ICD-10) codes F00-F03, G30, and G31, with a prior physician-confirmed diagnosis of dementia, were removed from the analysis (n = 5,350). Participants with incomplete data (n = 28,601) or a follow-up period of less than 30 days were also excluded (n = 157). After these exclusions, the final cohort for analysis consisted of 396,767 individuals, who were followed up until December 2019. Among them, 5,096 (1.3%) individuals were categorized into the VV group, while 391,671 (98.7%) comprised the non-VV group. To ensure a balanced comparison between these groups, a 1:5 propensity score matching (PSM) was performed, resulting in 5,092 individuals (16.7%) in the VV group and 25,460 (83.3%) in the non-VV group (**Fig 1**). The study adhered to the guidelines of the Declaration of Helsinki and was approved by the Institutional Review Board of Ewha Womans University Seoul Hospital (Institutional Review Board approval number: SEUMC 2023-05-020), which waived the requirement for informed consent due to the retrospective nature of the study and minimal risk of data collection to patients.

### Definition of varicose vein, outcomes and covariates

We established diagnoses of VV and all-cause dementia using ICD-10 codes. VV was defined as individuals diagnosed with VV on two or more occasions during the follow-up period, identified by the ICD-10 codes: I830-832, I839, or I868. Additionally, procedural codes for VV were defined according to ICD-10 diagnostic codes O0261-267, O2052, and O0215-217 [30,31]. The primary outcome of the study included all-cause dementia, including AD, VD, and other types of dementia, identified by the ICD-10 classification codes (F00-F03, G30, and G31) with antidementia drugs (donepezil, galantamine, rivastigmine, or memantine) [32,33]. Secondary outcomes considered occurrence of AD (F00 or G30) and VD (F01) [34,35]. This definition has gained wide acceptance due to its high accuracy, with a 94.7% positive predictive value [36].

Detailed definitions of the covariates are provided in S2 Appendix and in previous studies [37–39]. Data on variables such as age, sex, body mass index (BMI), household income, smoking status, alcohol consumption, physical activity, and comorbidities (hypertension, diabetes mellitus, dyslipidemia, stroke, myocardial infarction, chronic obstructive pulmonary disease, renal disease, liver disease, and cancer) were collected. The Charlson comorbidity index (CCI), a well-established tool, was used to assess comorbidities [39–41].

### Statistical analysis

To compare the VV and non-VV groups, continuous variables underwent independent t-tests, while categorical variables were assessed using Chi-squared tests (or Fisher’s exact test). PSM was initially conducted to mitigate confounding factors in observational studies. In this study, a 1:5 PSM ratio was employed between the VV and non-VV groups, with logistic regression analysis utilized to compute propensity scores in a logistic model. All covariates were incorporated into the propensity score model. The adequacy of PSM was evaluated using Standardized Mean Differences (SMDs), with PSM deemed appropriate when the absolute values of SMDs were less than 0.1. Additionally, we also conducted an analysis using a 1:3 matching ratio to consistently demonstrate statistically significant outcomes.

Kaplan–Meier survival curves, along with the log-rank test, were used to assess the association between VV and the occurrence of all-cause dementia, including AD and VD. After confirming adequate balance between the two groups, we performed Cox proportional hazards (CPH) regression analysis, presenting hazard ratios (HRs) with 95% confidence intervals (CIs) to assess the impact of VV on the occurrence of all-cause dementia. Data from PSM were further analyzed using Stratified Proportional Hazard models (SPH) to enhance estimation accuracy, considering the characteristics of paired matching data. The assumption of proportional hazards for covariates in CPH or SPH was evaluated using the Grambsch and Therneau test of scaled Schoenfeld residuals, which yielded satisfactory results. Multivariate survival models were adjusted for all potential confounding factors. All covariates were used to calculate it were included in a different model to avoid multicollinearity. It was confirmed that there was no multicollinearity in all multivariable models, with a variance inflation factor of < 2.0. Subgroup analysis was performed to evaluate association between presence and treatment of VV and the occurrence of AD and VD, respectively. Sensitivity analyses were performed to assess the risk of all-cause dementia between VV and non-VV groups for each categorical covariate using CPH and SPH, visualized using Forest plots. For sensitivity analysis, we performed additional analyses using Fine and Gray’s competing risk model. All statistical analyses were conducted using SAS 9.4 version (SAS Inc., Cary, NC, USA) and R software, version 4.2.1 (R Foundation for Statistical Computing, Vienna, Austria). The level of statistical significance for all tests was set at a two-sided p-value < 0.05.

## Results

### Baseline characteristics of participants

The baseline characteristics of the entire cohort (n = 396,767) are displayed in **Table 1**. The mean age of the total participants was 56.1 ± 9.3 year and 182,347 (46.0%) were males. The proportion of males was higher in the VV group compared to the non-VV group (57.0% vs 45.8%, *p <* 0.001), and the average BMI was higher in the VV group than the non-VV group (24.1 ± 2.8 kg/m^2^ vs. 24.0 ± 2.9 kg/m^2^, *p <* 0.001). The non-VV group exhibited a higher frequency of current smoking, alcohol consumption, and a history of diabetes mellitus compared with VV group. In contrast, VV group had more frequently history of comorbidities including dyslipidemia, chronic obstructive pulmonary disease, renal disease, liver disease, and cancer compared to non-VV group. After PSM among patients diagnosed with all-cause dementia, no major imbalances in baseline covariates between the two groups were observed, as assessed by SMD (SMD all < 0.1; Table 1).

**Table 1:** Baseline characteristics of study participants.

	Before PSM				After PSM 1:5		
Variable	Total	Varicose vein (-)	Varicose vein (+)	p-value	Varicose vein (-)	Varicose vein (+)	SMD*
		Mean ± SD, N (%)	Mean ± SD, N (%)		Mean ± SD, N (%)	Mean ± SD, N (%)	
Number	396,767	391,671	5,096		25,460	5,092	
Age, years	56.1 ± 9.3	56.1 ± 9.3	56.9 ± 8.2	<.001	56.8 ± 9.3	56.9 ± 8.2	-0.005
Sex				<.001			-0.012
Female	214,420 (54.0)	212,231 (54.2)	2,189 (43.0)		10,781 (42.3)	2,186 (42.9)	
Male	182,347 (46.0)	179,440 (45.8)	2,907 (57.0)		14,679 (57.7)	2,906 (57.1)	
Body mass index (kg/m^2^)	24.0 ± 2.9	24.0 ± 2.9	24.1 ± 2.8	<.001	24.1 ± 2.9	24.1 ± 2.8	-0.007
Household income				<.001			-0.008
First tertile, lowest	114,924 (29.0)	113,583 (29.0)	1,341 (26.3)		6,614 (26.0)	1,341 (26.3)	
Second tertile	144,163 (36.3)	142,243 (36.3)	1,920 (37.7)		9,620 (37.8)	1,917 (37.7)	
Third tertile, highest	137,680 (34.7)	135,845 (34.7)	1,835 (36.0)		9,226 (36.2)	1,834 (36.0)	
Smoking status				<.001			0.011
Never	283,324 (71.4)	279,333 (71.3)	3,991 (78.3)		20,057 (78.8)	3,988 (78.3)	
Former	37,154 (9.4)	36,614 (9.4)	540 (10.6)		2,654 (10.4)	540 (10.6)	
Current	76,289 (19.2)	75,724 (19.3)	565 (11.1)		2,749 (10.8)	564 (11.1)	
Alcohol consumption (days/week)				<.001			-0.002
None	239,231 (60.3)	235,924 (60.2)	3,307 (64.9)		16,510 (64.9)	3,306 (64.9)	
1-2 times	116,489 (29.4)	115,161 (29.4)	1,328 (26.1)		6,684 (26.3)	1,327 (26.1)	
3-4 times	25,683 (6.5)	25,379 (6.5)	304 (6.0)		1,476 (5.8)	303 (6.0)	
≥5 times	15,364 (3.8)	15,207 (3.9)	157 (3.0)		790 (3.1)	156 (3.0)	
Regular physical activity (days/week)				<.001			0.005
None	194,648 (49.1)	192,653 (49.2)	1,995 (39.2)		10,037 (39.4)	1,995 (39.2)	
1-4 days	153,646 (38.7)	151,592 (38.7)	2,054 (40.3)		10,300 (40.5)	2,053 (40.3)	
≥5 days	48,473 (12.2)	47,426 (12.1)	1,047 (20.5)		5,123 (20.1)	1,044 (20.5)	
Comorbidities							
Hypertension	97,212 (24.5)	95,978 (24.5)	1,234 (24.2)	0.633	6,024 (23.7)	1,234 (24.2)	-0.013
Diabetes mellitus	42,696 (10.8)	42,258 (10.8)	438 (8.6)	<.001	2,130 (8.4)	438 (8.6)	-0.008
Dyslipidemia	108,565 (27.4)	106,540 (27.2)	2,025 (39.7)	<.001	10,041 (39.4)	2,025 (39.8)	-0.007
Stroke	2,121 (0.5)	2,090 (0.5)	31 (0.6)	0.467	145 (0.6)	31 (0.6)	-0.005
Myocardial Infarction	968 (0.2)	952 (0.2)	16 (0.3)	0.308	74 (0.3)	16 (0.3)	-0.004
COPD	121,999 (30.8)	119,876 (30.6)	2,123 (41.7)	<.001	10,624 (41.7)	2,123 (41.7)	0.000
Renal disease	15,451 (3.9)	15,186 (3.9)	265 (5.2)	<.001	1,272 (5.0)	265 (5.2)	-0.009
Liver disease	92,522 (23.3)	90,840 (23.2)	1,682 (33.0)	<.001	8,295 (32.6)	1,682 (33.0)	-0.001
Cancer	22,266 (5.6)	21,874 (5.6)	392 (7.7)	<.001	1,853 (7.3)	392 (7.7)	-0.016
Charlson comorbidity index				0.393			0.013
0	366,563 (93.4)	361,781 (93.4)	4,782 (93.9)		23,987 (94.2)	4,782 (93.9)	
1	23,452 (6.0)	23,169 (6.0)	283 (5.6)		1,361 (5.4)	283 (5.6)	
≥2	2,275 (0.6)	2,248 (0.6)	27 (0.5)		112 (0.4)	27 (0.5)	

COPD, chronic obstructive pulmonary disease; N, number; PSM, propensity score matching; SD, standard deviation; SMD, standardized mean difference.

* All standardized mean difference values were <0.1 in the propensity score matched cohort.

### Association of presence of varicose vein with dementia

**Fig 2:**
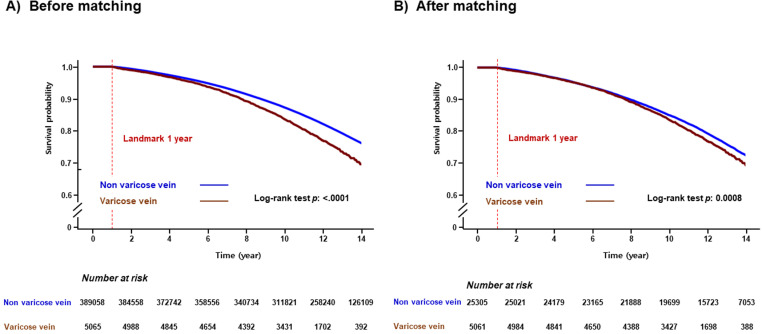
Kaplan-Meier survival curves of all-cause dementia according to varicose vein before propensity score matching (A) and after propensity score matching (B).

During a median follow-up of 13.33 (interquartile range 10.4–16.26) years, 55,329 (13.9%) cases of all-cause dementia, 38,673 (9.7%) cases of AD, and 15,013 (3.7%) cases of VD had occurred. The Kaplan–Meier survival curves for the occurrence of all-cause dementia according to presence of VV were shown in Fig 2A and 2B. In the multivariate analysis, VV group consistently showed increased risk of all-cause dementia compared to non-VV group before PSM (HR: 1.241, 95% CI: 1.170–1.316, p < 0.001) and after PSM (HR: 1.235, 95% CI: 1.147–1.329, p < 0.001; Table 2, S1 Table). Regarding subgroups of dementia, presence of VV was not associated with each AD and VD in multivariable analysis (Table 2, S2 and S3 Tables).

**Table 2:** Results of Cox regression analysis for the association of varicose vein with incidence risk of dementia.

Variable	Before PSM N = 396,767			After PSM 1:5 N = 30,552		
	Incidence rate (per 100,000 person-years)	Crude HR (95%CI)	Adjusted HR (95%CI)	Incidence rate (per 100,000 person-years)	Crude HR (95%CI)	Adjusted HR (95%CI)
All-cause dementia	1,820.128	1.309 (1.234-1.388)	1.241 (1.170-1.316)	2,103.471	1.133 (1.060-1.211)	1.235 (1.147-1.329)
Alzheimer’s disease	794.212	1.000 (0.909-1.100)	1.004 (0.912-1.104)	889.003	1.021 (1.002-1.040)	1.020 (0.899-1.158)
Vascular dementia	302.086	1.253 (0.915-1.591)	1.185 (0.802-1.568)	322.922	1.232 (1.065-1.398)	1.023 (0.848-1.557)

Abbreviations: CI, confidence interval; HR, hazard ratio; N, number; PSM, propensity score matching. Values from multivariate Cox regression models adjusted for age, sex, body mass index, household income, smoking status, alcohol consumption, regular physical activity, comorbidities, and Charlson comorbidity index.

**Fig 3:**
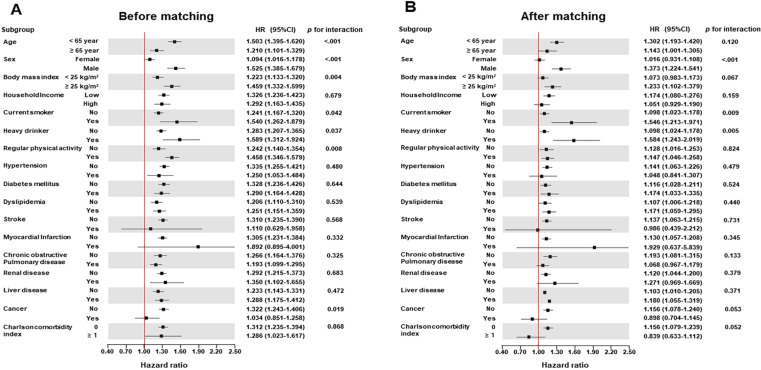
Forest plots of occurrence of all-cause dementia for the varicose vein according to demographic data and comorbidities.

In sensitivity analysis, after PSM, the association of presence of VV with the incidence risk of all-cause dementia was consistently noted regardless of covariates, furthermore, men, current smokers, and heavy drinker (≥3 days/week) were more significantly related with the incidence risk of all-cause dementia compared to women, non-current smokers, and non-heavy drinker, respectively (Fig 3). Considering landmark analysis, the relationship of presence of VV with the incidence risk of all-cause dementia was also consistently demonstrated before PSM (HR: 1.250, 95% CI: 1.178–1.326, p < 0.001) and after PSM (HR: 1.218, 95% CI: 1.131–1.312, p = 0.041; S4 Table and S2 Fig). Additionally, we also conducted an analysis using a 1:3 matching ratio, which produced similar results to the 1:5 matching, consistently demonstrating statistically significant outcomes (HR [95% CI]: 1.220 [1.140–1.305]; S5 Table). In sensitivity analyses using the competing risk model, the association between the presence of VV and the incidence risk of dementia remained consistent (S6 Table).

### Association of treatment/procedure of varicose vein with dementia

Detailed frequency of treatment/procedure information was provided in S7 Table. In contrast, considering the association of treatment/procedure of VV, incidence risk of all-cause dementia was not different between with treatment/procedure of VV group and without treatment/procedure of VV group before PSM (HR: 0.884, 95% CI: 0.778–1.005) and after PSM (HR: 0.920, 95% CI: 0.796–1.062). Regarding incidence risk of AD, treatment/procedure of VV group was associated with decreased risk of AD before PSM (HR: 0.778, 95% CI: 0.624–0.970), but not associated with decreased risk of AD after PSM (HR: 0.787, 95% CI: 0.615–1.007). In contrast, treatment/procedure of VV group was significantly associated with decreased risk of VD before PSM (HR: 0.630, 95% CI: 0.437–0.908) and after PSM (HR: 0.566, 95% CI: 0.382–0.841; Table 3, S8-S11 Tables). In sensitivity analyses using the competing risk model, the association between treatment/procedure for VV and the incidence risk of dementia remained consistent (S12 Table).

**Table 3:** Results of Cox regression analysis for the association of treatment/procedure for varicose vein with incidence risk of dementia.

Variable	Before PSM N = 5,096			After PSM 1:1 N = 3,882		
	Incidence rate (per 100,000 person-years)	Crude HR (95%CI)	Adjusted HR (95%CI)	Incidence rate (per 100,000 person-years)	Crude HR (95%CI)	Adjusted HR (95%CI)
All-cause dementia	2,114.395	0.735 (0.648-0.834)	0.884 (0.778-1.005)	1,836.783	0.900 (0.779-1.038)	0.920 (0.796-1.062)
Alzheimer’s disease	763.989	0.565 (0.455-0.701)	0.778 (0.624-0.970)	617.014	0.731 (0.572-0.934)	0.787 (0.615-1.007)
Vascular dementia	291.892	0.505 (0.354-0.721)	0.630 (0.437-0.908)	258.834	0.527 (0.357-0.780)	0.566 (0.382-0.841)

Abbreviations: CI, confidence interval; HR, hazard ratio; N, number; PSM, propensity score matching. Values from multivariate Cox regression models adjusted for age, sex, body mass index, household income, smoking status, alcohol consumption, regular physical activity, comorbidities, and Charlson comorbidity index.

## Discussion

In this nationwide population-based cohort study, we found that patients diagnosed with VV had an increased risk of all-cause dementia, especially among male, current smokers, and heavy drinker. We also found population with treatment/procedure VV had lower occurrence of VD than untreated cases.

There is growing interest in the function of veins and their relationship with neurodegenerative diseases, including dementia. Previous study has suggested that the presence of VV is a significant risk factor for decreased scores on the Mini-Mental State Examination and the Montreal Cognitive Assessment over a two-year period [42]. Peripheral artery disease, which may be related to the presence of VV, is associated with an increased risk of VD [43,44]. Additionally, VV could be indicative of cerebral venous insufficiency. The cerebral venous system, which contains approximately 70% of the total cerebral blood volume, is crucial for maintaining brain tissue homeostasis [45]. Several research have suggested that cerebral venous insufficiency affects certain central nervous system disorders, including AD [46,47], parkinson’s disease [48], transient global amnesia [49]. Moreover, previous studies have shown that extracranial venous abnormalities, which can be potential sources of venous hypertension, are related to hypoperfusion in the brain parenchyma and cerebral small vessel disease, including white matter hyperintensities and cerebral microbleeds, leading to dementia [50,51]. However, longitudinal cohort studies providing data to investigate the association between VV and subsequent dementia incidence are currently limited [24]. In line with previous studies, we showed that patients diagnosed with VV had an increased incidence of all-cause dementia.

VV might induce inflammation and endothelial dysfunction [12,52]. While the hallmark pathologies of AD include the progressive accumulation of the protein fragment beta-amyloid, long-term systemic inflammation may also play a key role in AD. Peripheral inflammation has been linked to central nervous system inflammation, with inflammatory molecules being associated with microglial activation, leading to AD [53,54]. Consistent with our findings, a previous study demonstrated that individuals with VV had a higher incidence of AD compared to the control group, and that those with complicated VV experienced a higher incidence of AD than those with uncomplicated VV [24]. Although VD results from cerebrovascular disease, studies have indicated that chronically elevated high sensitivity C-reactive protein (CRP) and the combination of elevated high sensitivity CRP and interleukin-6 due to inflammation are associated with an increased rate of VD [55,56]. Also, level of Vascular cell adhesion protein 1 was associated with the severity of white matter changes, poor short-term memory, and visuospatial function [57]. Previous study has shown an association between VV and cerebral autosomal dominant arteriopathy with subcortical infarcts and leukoencephalopathy, leading to VD [58]. Consequently, systemic inflammation and endothelial dysfunction could be related to cognitive function disturbances [59]. However, subgroup analysis of present study showed that presence of VV was not associated with each AD and VD in multivariable analysis. This may be because a large proportion of patients with pathology of VD coexists with Alzheimer’s pathology, and inflammation and endothelial dysfunction are not the primary causes of AD and VD. However, due to the small portion of VD (3.7%) and AD (9.7%) patients included in the current study and the low proportion of patients diagnosed with VV (1.3%), further studies are required to address this issue. In other words, the statistical power was insufficient due to the small sample size, so the association of VV with AD and VD may not have been significant. In addition, there are other subtypes of dementia other than AD and VD, but since the NHIS dataset does not provide accurate information on this, further studies should be needed to address these issues.

In present study, treatment/procedure of VV group was significantly associated with decreased risk of VD. During the recent years, many studies have demonstrated tumor necrosis factor-α inhibitors may be beneficial to wound healing in chronic venous legs ulcers as well as improving cognitive function in VD patients [60,61]. Also, because VV are at a risk of cardiovascular events, treatment/procedure of VV group decrease risk of cerebrovascular disease leading to VD [62]. Because chronic venous insufficiency altered venous drainage of the brain, leading to cerebral small vessel disease, including white matter hyperintensities and cerebral microinfarcts, which associated with VD [63,64], treatment/procedure of VV group might be decreased risk of VD.

Our study has several limitations. First, although the severity of VV cannot be presented in health claim data, we performed additional analysis regarding population received treatment/procedure for VV. Nevertheless, our analysis did not include categorization based on severity of VV. Second, apart from the adjusted covariates in our study, unmeasured confounders including education, family history of dementia, genetic predisposition, different levels of urbanization, medical facilities, and monthly insured premiums could potentially influence the outcomes. Third, cognitive assessment, laboratory examination, and imaging studies were not available in the claims data. Fourth, our dataset comprises a random sampling of 10% of all South Koreans and thus might not fully represent the entire South Korean population. Additionally, due to the specific nature of the NHIS-HEALS dataset, which consists solely of Koreans, the results of this study may be biased racially, limiting the broader applicability of the conclusions to other demographic groups. Therefore, it’s crucial to carry out additional research encompassing diverse racial and ethnic populations. Fifth, since the NHIS-HEALS database targets individuals aged 40 years or older, we could not confirm the association between VV and dementia in the population under 40 years of age. Finally, the retrospective nature of the study presents challenges in establishing cause-and-effect relationships.

## Conclusions

Our study showed that presence of VV was associated with increased risk of all-cause dementia, and treatment/procedure of VV was likely to reduce the incidence risk of VD. In conclusion, our study suggests that the presence of VV may be associated with an increased risk of future all-cause dementia.

## Supporting information

S1 Appendix Supplementary Methods 1. Data source.
(DOCX)

S2 Appendix Supplementary Methods 2. Definition of covariates.
(DOCX)

S1 Table Results of Cox regression analysis for the association of varicose vein with risk of all-cause dementia. (DOCX)

S2 Table Results of Cox regression analysis for the association of varicose vein with risk of Alzheimer’s disease. (DOCX)

S3 Table Results of Cox regression analysis for the association of varicose vein with risk of vascular dementia. (DOCX)

S4 Table Results of Cox regression analysis for the association of varicose vein with incidence risk of dementia: A 1-year landmark analysis. (DOCX)

S5 Table Results of Cox regression analysis for the association of varicose vein with incidence risk of dementia in a 1:3 Matched Cohort. (DOCX)

S6 Table Results of Fine and Gray competing risk regression analysis for the association of varicose veins with incidence risk of dementia. (DOCX)

S7 Table Frequency table of procedure code for population who received procedure/treatment for varicose vein. (DOCX)

S8 Table Results of Cox regression analysis for the association of procedure/treatment for varicose vein with risk of all-cause dementia. (DOCX)

S9 Table Results of Cox regression analysis for the association of procedure/treatment for varicose vein with risk of Alzheimer’s disease. (DOCX)

S10 Table Results of Cox regression analysis for the association of procedure/treatment for varicose vein with risk of vascular dementia. (DOCX)

S11 Table Results of Cox regression analysis for the association of procedure/treatment for varicose vein with incidence risk of dementia: A 1 - year landmark analysis. (DOCX)

S12 Table Results of Fine and Gray competing risk regression analysis for the association of procedure/treatment for varicose vein with incidence risk of dementia. (DOCX)

S1 Fig Geographical distribution of this database. (PDF)

S2 Fig Kaplan-Meier survival curves of all-cause dementia according to varicose vein before propensity score matching (A) and after propensity score matching (B): A 1-year landmark analysis. (PDF)
