# Supplementary material for: Association between varicose veins and occurrence of dementia: A nationwide population-based cohort study
Source: PLoS One. 2025 Apr 30;20(4):e0322892. doi: 10.1371/journal.pone.0322892 (PMC12043132; doi:10.1371/journal.pone.0322892)
Supplement: S1 Appendix — Data source. (DOCX) [file pone.0322892.s001.docx]

**Supplementary Methods 1.** Data source

The National Health Insurance Service (NHIS) is a mandatory social insurance system in South Korea that provides coverage for medical expenses. Given that approximately 97% of the South Korean population is covered by the NHIS, its database offers valuable insights into medical trends and practices within the country. However, access to and use of the NHIS database for research purposes is restricted to safeguard personal identifiable information and due to the large volume of data. To address these concerns, the NHIS has established the National Health Screening (HEALS) cohort. The NHIS-Health Screening Cohort (NHIS-HEALS) consists of individuals who participated in the health screening programs offered by the NHIS in South Korea. The general health screening program is available to all adults aged 40 years or older at least once every two years. The NHIS-HEALS cohort includes a randomly selected sample of 10% (n = 514,866) of participants [1].

**Reference**

1. Seong SC, Kim YY, Park SK, Khang YH, Kim HC, Park JH, et al. Cohort profile: the National Health Insurance Service-National Health Screening Cohort (NHIS-HEALS) in Korea. BMJ open. 2017;7(9):e016640. Epub 2017/09/28. doi: 10.1136/bmjopen-2017-016640. PubMed PMID: 28947447; PubMed Central PMCID: PMCPMC5623538.
