# Supplementary material for: Association between varicose veins and occurrence of dementia: A nationwide population-based cohort study
Source: PLoS One. 2025 Apr 30;20(4):e0322892. doi: 10.1371/journal.pone.0322892 (PMC12043132; doi:10.1371/journal.pone.0322892)
Supplement: S2 Appendix — Definition of covariates. (DOCX) [file pone.0322892.s002.docx]

**Supplementary Methods 2.** Definition of covariates

*Smoking status*

Smoking status was used to categorize participants into three groups: none, former smoker, and current smoker. Current smoker was classified according to the WHO definition as a person who has smoked more than five packs (100 cigarettes) in a lifetime and smoked daily or occasionally for the last 28 days. Former smoker was defined as a person who had smoked more than 100 cigarettes in a lifetime and had not smoked in the last 28 days [1].

*Alcohol consumption*

Alcohol consumption was categorized into two groups: <3 days per week and ≥3 days per week [2].

*Regular physical activity*

Regular physical activity was categorized into two groups: <3 days per week and ≥3 days per week [3].

*Hypertension*

Hypertension was defined as using at least one claim of International Classification of Diseases, Tenth Revision (ICD-10) code (I10–15) with the prescription of an anti-hypertensive agent, claims of ICD-10 code (I10–15) more than two times, a systolic blood pressure of ≥140 mmHg and a diastolic blood pressure of ≥90 mmHg or positive checking in self-report questionnaire on hypertension in the health examination program [4].

*Diabetes mellitus*

Diabetes mellitus was as defined using at least one claim of ICD-10 code (E11–14) with the prescription of an anti-diabetic agent, claims of ICD-10 code (E11–14) more than two times, fasting serum glucose concentration of ≥7.0 mmol/L or positive checking in self-report questionnaire on diabetes mellitus in the health examination program [4].

*Dyslipidemia*

Dyslipidemia was defined as using at least one claim of ICD-10 code (E78) with the prescription of a lipid lowing agent, claims of ICD-10 code (E78) more than two times or total cholesterol level of ≥240 mg/dL [4].

*Stroke*

Stroke was defined as two or more claims of ICD-10 codes (I60–64) with brain CT/MRI and hospital admission [4].

*Myocardial infarction*

Myocardial infarction was diagnosed using ICD-10 codes I21, I22, with more than one diagnosis during admission or at outpatient clinics [4].

*Chronic obstructive pulmonary disease*

COPD patients were identified herein based on the ICD-10 codes (J41–J44), completion at least two treatment courses, or at least one hospitalization during the current or preceding year [5].

*Renal disease*

Renal disease was defined as using claims of ICD–10 codes (N17-19, I12-13, E08.2, E10.2, E11.2, E13.2) more than two times or estimated glomerular filtration rate of <60 mL/min/1.73m^2^ [6].

*Liver disease*

Patients with liver disease were defined as those who had chronic hepatitis (ICD-10 code B18), HBV (ICD-10 code B18.0, B18.1), HCV (ICD-10 code: B18.2), cirrhosis (ICD-10 codes K70.2, K70.3, K74.x, and K71.7), or other liver diseases including alcoholic liver disease (ICD-10 codes K70, K70.1, K70.9, K76.1, K76.5, K76.6, and Z22.5) [7].

*Cancer*

Cancer was defined as using claims of ICD-10 code (C00–C97) more than two times with cancer specific deductible code (V027, V193-4) from the Health Insurance Review and Assessment Service [8].

*Charlson comorbidity index*

The Charlson comorbidity index score was calculated for each subject based on diseases diagnosed before index date and divided into three groups (0, 1, and ≥2 scores) [9].

**Reference**

1. Kim C-Y, Lee CM, Lee S, Yoo JE, Lee H, Park HE, et al. The Association of Smoking Status and Clustering of Obesity and Depression on the Risk of Early–Onset Cardiovascular Disease in Young Adults: A Nationwide Cohort Study. kcj. 2022;53(1):17-30. doi: 10.4070/kcj.2022.0179.

2. Son J-W, Lee S, Kang M, Shin Y-A, Kim J-H. Optimal Frequency Intensity of Physical Activity to Reduce the Risk of Hypertension in the Korean Population. Exerc Sci. 2022;31(1):129-40. doi: 10.15857/ksep.2021.00626.

3. Han S, Jang H-D, Choi S, Kim GD, Han K, Lim H, et al. Changes in physical activity and risk of fracture: a Korean nationwide population-based cohort study. Scientific Reports. 2020;10(1):16266. doi: 10.1038/s41598-020-73495-1.

4. Choi EK. Cardiovascular Research Using the Korean National Health Information Database. Korean circulation journal. 2020;50(9):754-72. Epub 2020/07/30. doi: 10.4070/kcj.2020.0171. PubMed PMID: 32725984; PubMed Central PMCID: PMCPMC7441000.

5. Min J, Park JE, Kim SY, Kim YY, Park JH. Growing disparity in the prevalence of chronic obstructive pulmonary disease between people with and without disabilities: a Korean nationwide serial cross-sectional study. Sci Rep. 2023;13(1):13205. Epub 2023/08/15. doi: 10.1038/s41598-023-39319-8. PubMed PMID: 37580327; PubMed Central PMCID: PMCPMC10425333.

6. Kim J, Jeon J, Lee HS, Lee KY. Association Between the Risk for Cardiovascular Events and Antiviral Treatment for Herpes Zoster. Clinical infectious diseases : an official publication of the Infectious Diseases Society of America. 2021;73(5):758-64. Epub 2020/09/15. doi: 10.1093/cid/ciaa1384. PubMed PMID: 32926085.

7. Suh JK, Lee J, Lee J-H, Shin S, Tchoe Hj, Kwon J-W. Risk factors for developing liver cancer in people with and without liver disease. PLOS ONE. 2018;13(10):e0206374. doi: 10.1371/journal.pone.0206374.

8. Lim H, Lee Y-H, Bae S, Koh D-H, Yoon M, Lee B-E, et al. Cancer cluster among small village residents near the fertilizer plant in Korea. PloS one. 2021;16(2):e0247661. doi: 10.1371/journal.pone.0247661.

9. Kwon HS, Suh J, Kim M-h, Yoo B, Han M, Koh I-S, et al. Five-Year Community Management Rate for Dementia Patients: A Proposed Indicator for Dementia Policies. J Clin Neurol. 2022;18(1):24-32.
