# Supplementary material for: Association between varicose veins and occurrence of dementia: A nationwide population-based cohort study
Source: PLoS One. 2025 Apr 30;20(4):e0322892. doi: 10.1371/journal.pone.0322892 (PMC12043132; doi:10.1371/journal.pone.0322892)
Supplement: S1 Table — (DOCX) [file pone.0322892.s003.docx]

**S1 Table.** Results of Cox regression analysis for the association of varicose vein with risk of all-cause dementia.

| Variables | Before PSM  N = 396,767 | After PSM 1:5 N = 30,552 |
| --- | --- | --- |
|  | Adjusted  HR (95% CI) | Adjusted  HR (95% CI) |
| Without varicose vein | Reference | Reference |
| With varicose vein | 1.241 (1.170 - 1.316) | 1.235 (1.147 - 1.329) |
| Age, years | 1.100 (1.099 - 1.100) | 1.100 (1.096 - 1.104) |
| Sex |  |  |
| Male | Reference | Reference |
| Female | 1.465 (1.440 - 1.490) | 1.529 (1.414 - 1.652) |
| Body mass index (kg/m^2^) | 0.997 (0.995 - 1.000) | 1.005 (0.995 - 1.015) |
| Household income |  |  |
| Low | Reference | Reference |
| Middle | 0.978 (0.962 - 0.993) | 0.991 (0.922 - 1.066) |
| High | 0.875 (0.860 - 0.890) | 0.894 (0.829 - 0.964) |
| Smoking status |  |  |
| Never | Reference | Reference |
| Former | 1.007 (0.978 - 1.036) | 1.016 (0.901 - 1.146) |
| Current | 1.042 (1.018 - 1.066) | 1.030 (0.913 - 1.162) |
| Alcohol consumption (days/week) |  |  |
| None | Reference | Reference |
| 1 - 2 times | 0.935 (0.917 - 0.953) | 0.982 (0.905 - 1.066) |
| 3 - 4 times | 1.007 (0.973 - 1.042) | 1.091 (0.942 - 1.264) |
| ≥ 5 times | 1.162 (1.122 - 1.203) | 1.099 (0.922 - 1.310) |
| Regular physical activity (days/week) |  |  |
| None | Reference | Reference |
| 1 - 4 days | 0.875 (0.861 - 0.889) | 0.881 (0.822 - 0.944) |
| ≥ 5 days | 0.926 (0.908 - 0.945) | 0.892 (0.822 - 0.969) |
| Comorbidities |  |  |
| Hypertension | 1.070 (1.054 - 1.087) | 1.134 (1.060 - 1.215) |
| Diabetes mellitus | 1.197 (1.174 - 1.221) | 1.190 (1.078 - 1.314) |
| Dyslipidemia | 1.148 (1.130 - 1.166) | 1.179 (1.103 - 1.261) |
| Stroke | 1.793 (1.683 - 1.910) | 1.865 (1.366 - 2.548) |
| Myocardial Infarction | 1.049 (0.935 - 1.178) | 1.184 (0.711 - 1.972) |
| COPD | 1.215 (1.198 - 1.232) | 1.247 (1.174 - 1.325) |
| Renal disease | 1.053 (1.022 - 1.084) | 0.997 (0.882 - 1.127) |
| Liver disease | 1.239 (1.219 - 1.259) | 1.215 (1.136 - 1.300) |
| Cancer | 1.143 (1.114 - 1.173) | 1.161 (1.045 - 1.291) |
| Charlson comorbidity index |  |  |
| 0 | Reference | Reference |
| 1 | 1.044 (1.017 - 1.072) | 1.017 (0.898 - 1.151) |
| ≥ 2 | 1.012 (0.935 - 1.096) | 0.721 (0.463 - 1.124) |

Abbreviations: CI, confidence interval; COPD, chronic obstructive pulmonary disease; HR, hazard ratio; N, number; PSM, propensity score matching
