# Supplementary material for: Association between varicose veins and occurrence of dementia: A nationwide population-based cohort study
Source: PLoS One. 2025 Apr 30;20(4):e0322892. doi: 10.1371/journal.pone.0322892 (PMC12043132; doi:10.1371/journal.pone.0322892)
Supplement: S2 Table — (DOCX) [file pone.0322892.s004.docx]

**S2 Table.** Results of Cox regression analysis for the association of varicose vein with risk of Alzheimer’s disease.

| Variables | Before PSM  N = 396,767 | After PSM 1:5 N = 30,552 |
| --- | --- | --- |
|  | Adjusted  HR (95% CI) | Adjusted  HR (95% CI) |
| Without varicose vein | Reference | Reference |
| With varicose vein | 1.004 (0.912 - 1.104) | 1.020 (0.899 - 1.158) |
| Age, years | 1.143 (1.141 - 1.144) | 1.157 (1.150 - 1.165) |
| Sex |  |  |
| Male | Reference | Reference |
| Female | 1.364 (1.330 - 1.399) | 1.304 (1.152 - 1.477) |
| Body mass index (kg/m^2^) | 0.990 (0.986 - 0.993) | 1.012 (0.996 - 1.028) |
| Household income |  |  |
| Low | Reference | Reference |
| Middle | 0.968 (0.945 - 0.992) | 0.966 (0.857 - 1.088) |
| High | 0.885 (0.863 - 0.908) | 0.908 (0.803 - 1.027) |
| Smoking status |  |  |
| Never | Reference | Reference |
| Former | 1.009 (0.966 - 1.054) | 1.105 (0.908 - 1.344) |
| Current | 1.135 (1.097 - 1.175) | 1.103 (0.906 - 1.345) |
| Alcohol consumption (days/week) |  |  |
| None | Reference | Reference |
| 1 - 2 times | 0.904 (0.877 - 0.931) | 0.879 (0.763 - 1.012) |
| 3 - 4 times | 0.966 (0.916 - 1.019) | 0.861 (0.669 - 1.107) |
| ≥ 5 times | 1.123 (1.068 - 1.180) | 0.778 (0.584 - 1.036) |
| Regular physical activity (days/week) |  |  |
| None | Reference | Reference |
| 1 - 4 days | 0.838 (0.818 - 0.859) | 0.789 (0.703 - 0.885) |
| ≥ 5 days | 0.889 (0.863 - 0.916) | 0.814 (0.714 - 0.928) |
| Comorbidities |  |  |
| Hypertension | 1.049 (1.026 - 1.072) | 1.083 (0.973 - 1.206) |
| Diabetes mellitus | 1.321 (1.285 - 1.357) | 1.487 (1.277 - 1.731) |
| Dyslipidemia | 1.121 (1.096 - 1.148) | 1.117 (1.002 - 1.244) |
| Stroke | 1.724 (1.587 - 1.873) | 1.583 (1.025 - 2.444) |
| Myocardial Infarction | 1.027 (0.877 - 1.202) | 0.681 (0.317 - 1.463) |
| COPD | 1.168 (1.144 - 1.193) | 1.110 (1.006 - 1.226) |
| Renal disease | 1.092 (1.048 - 1.137) | 1.080 (0.894 - 1.304) |
| Liver disease | 1.175 (1.148 - 1.203) | 1.081 (0.969 - 1.206) |
| Cancer | 1.145 (1.105 - 1.187) | 1.095 (0.930 - 1.289) |
| Charlson comorbidity index |  |  |
| 0 | Reference | Reference |
| 1 | 1.038 (0.999 - 1.079) | 1.022 (0.834 - 1.253) |
| ≥ 2 | 1.129 (1.014 - 1.256) | 0.822 (0.433 - 1.558) |

Abbreviations: CI, confidence interval; COPD, chronic obstructive pulmonary disease; HR, hazard ratio; N, number; PSM, propensity score matching.
