# Supplementary material for: Association between varicose veins and occurrence of dementia: A nationwide population-based cohort study
Source: PLoS One. 2025 Apr 30;20(4):e0322892. doi: 10.1371/journal.pone.0322892 (PMC12043132; doi:10.1371/journal.pone.0322892)
Supplement: S3 Table — (DOCX) [file pone.0322892.s005.docx]

**S3 Table.** Results of Cox regression analysis for the association of varicose vein with risk of vascular dementia.

| Variables | Before PSM  N = 396,767 | After PSM 1:5 N = 30,552 |
| --- | --- | --- |
|  | Adjusted  HR (95% CI) | Adjusted  HR (95% CI) |
| Without varicose vein | Reference | Reference |
| With varicose vein | 1.185 (0.802 - 1.568) | 1.023 (0.848 - 1.207) |
| Age, years | 1.108 (1.106 - 1.110) | 1.120 (1.110 - 1.131) |
| Sex |  |  |
| Male | Reference | Reference |
| Female | 1.112 (1.069 - 1.157) | 1.101 (0.914 - 1.327) |
| Body mass index (kg/m^2^) | 0.999 (0.993 - 1.004) | 1.015 (0.991 - 1.040) |
| Household income |  |  |
| Low | Reference | Reference |
| Middle | 0.979 (0.942 - 1.018) | 0.874 (0.732 - 1.044) |
| High | 0.886 (0.851 - 0.923) | 0.798 (0.664 - 0.958) |
| Smoking status |  |  |
| Never | Reference | Reference |
| Former | 0.935 (0.874 - 1.001) | 0.935 (0.699 - 1.252) |
| Current | 1.119 (1.061 - 1.179) | 1.218 (0.917 - 1.618) |
| Alcohol consumption (days/week) |  |  |
| None | Reference | Reference |
| 1 - 2 times | 0.953 (0.910 - 0.999) | 1.074 (0.876 - 1.317) |
| 3 - 4 times | 1.016 (0.938 - 1.101) | 1.065 (0.745 - 1.522) |
| ≥ 5 times | 1.122 (1.038 - 1.214) | 1.472 (0.975 - 2.220) |
| Regular physical activity (days/week) |  |  |
| None | Reference | Reference |
| 1 - 4 days | 0.827 (0.796 - 0.860) | 0.888 (0.747 - 1.057) |
| ≥ 5 days | 0.877 (0.836 - 0.920) | 0.939 (0.774 - 1.138) |
| Comorbidities |  |  |
| Hypertension | 1.214 (1.172 - 1.256) | 1.253 (1.071 - 1.467) |
| Diabetes mellitus | 1.355 (1.298 - 1.415) | 1.281 (1.022 - 1.607) |
| Dyslipidemia | 1.174 (1.131 - 1.218) | 1.333 (1.132 - 1.570) |
| Stroke | 2.412 (2.160 - 2.695) | 2.733 (1.488 - 5.018) |
| Myocardial Infarction | 1.023 (0.800 - 1.309) | 1.072 (0.305 - 3.775) |
| COPD | 1.117 (1.080 - 1.155) | 0.983 (0.847 - 1.139) |
| Renal disease | 1.178 (1.107 - 1.255) | 1.117 (0.852 - 1.463) |
| Liver disease | 1.111 (1.070 - 1.154) | 1.145 (0.975 - 1.344) |
| Cancer | 1.138 (1.074 - 1.206) | 1.014 (0.793 - 1.297) |
| Charlson comorbidity index |  |  |
| 0 | Reference | Reference |
| 1 | 1.053 (0.990 - 1.120) | 0.909 (0.671 - 1.230) |
| ≥ 2 | 1.117 (0.943 - 1.324) | 0.934 (0.375 - 2.325) |

Abbreviations: CI, confidence interval; COPD, chronic obstructive pulmonary disease; HR, hazard ratio; N, number; PSM, propensity score matching.
