# Supplementary material for: Association between varicose veins and occurrence of dementia: A nationwide population-based cohort study
Source: PLoS One. 2025 Apr 30;20(4):e0322892. doi: 10.1371/journal.pone.0322892 (PMC12043132; doi:10.1371/journal.pone.0322892)
Supplement: S4 Table — (DOCX) [file pone.0322892.s006.docx]

**S4 Table.** Results of Cox regression analysis for the association of varicose vein with incidence risk of dementia: A 1-year landmark analysis.

| Variable | Before PSM,  N = 394,123 | | | After PSM 1:1, N = 30,366 | | |
| --- | --- | --- | --- | --- | --- | --- |
|  | Incidence rate  (per 100,000 person - years) | Crude  HR (95% CI) | Adjusted  HR (95% CI) | Incidence rate  (per 100,000 person - years) | Crude  HR (95% CI) | Adjusted  HR (95% CI) |
| All - cause dementia | 1,778.017 | 1.317 (1.241 - 1.397) | 1.250 (1.178 - 1.326) | 2,065.665 | 1.121 (1.048 - 1.199) | 1.218 (1.131 - 1.312) |
| Alzheimer’s disease | 772.281 | 1.000 (0.908 - 1.102) | 1.012 (0.918 - 1.115) | 848.061 | 1.018 (1.003 - 1.034) | 1.016 (0.906 - 1.125) |
| Vascular dementia | 289.552 | 1.235 (0.915-1.556) | 1.170 (0.802 - 1.541) | 304.997 | 1.224 (1.065 - 1.343) | 1.123 (0.943 - 1.304) |

Abbreviations: CI, confidence interval; HR, hazard ratio; N, number; PSM, propensity score matching. Values from multivariate Cox regression models adjusted for age, sex, body mass index, household income, smoking status, alcohol consumption, regular physical activity, comorbidities, and Charlson comorbidity index.
