# Supplementary material for: Association between varicose veins and occurrence of dementia: A nationwide population-based cohort study
Source: PLoS One. 2025 Apr 30;20(4):e0322892. doi: 10.1371/journal.pone.0322892 (PMC12043132; doi:10.1371/journal.pone.0322892)
Supplement: S5 Table — (DOCX) [file pone.0322892.s007.docx]

**S5 Table.** Results of Cox regression analysis for the association of varicose vein with incidence risk of dementia in a 1:3 Matched Cohort.

| Variable | After PSM 1:3 N = 20,368 | | | |
| --- | --- | --- | --- | --- |
|  | Incidence rate  (per 100,000 person - years) | Crude  HR (95% CI) | Adjusted-1  HR (95% CI) | Adjusted-2  HR (95% CI) |
| All - cause dementia | 2,118.989 | 1.309 (1.234 - 1.388) | 1.220 (1.140 - 1.305) | 1.217 (1.137 - 1.301) |
| Alzheimer’s disease | 881.806 | 1.002 (0.911 - 1.095) | 1.003 (0.913 - 1.094) | 1.002 (0.912 - 1.093) |
| Vascular dementia | 311.206 | 1.251 (0.913 - 1.589) | 1.232 (0.915 - 1.549) | 1.229 (0.917 - 1.538) |

Abbreviations: CI, confidence interval; HR, hazard ratio; N, number; PSM, propensity score matching. Adjusted-1 HR represents the hazard ratio adjusted for age, sex, body mass index, household income, smoking status, alcohol consumption, regular physical activity, comorbidities, and the Charlson comorbidity index. Adjusted-2 HR represents the hazard ratio further adjusted for social determinants of health in addition to the variables included in Adjusted-1 HR.
