# Supplementary material for: Association between varicose veins and occurrence of dementia: A nationwide population-based cohort study
Source: PLoS One. 2025 Apr 30;20(4):e0322892. doi: 10.1371/journal.pone.0322892 (PMC12043132; doi:10.1371/journal.pone.0322892)
Supplement: S6 Table — (DOCX) [file pone.0322892.s008.docx]

**S6 Table.** Results of Fine and Gray competing risk regression analysis for the association of varicose veins with incidence risk of dementia.

| Variable | Before PSM | After 1:5 PSM |
| --- | --- | --- |
|  | N = 396,767 | N = 30,552 |
|  | Adjusted | Adjusted |
|  | HR (95%CI) | HR (95%CI) |
| All-cause dementia | 1.219 (1.147 - 1.295) | 1.205 (1.129 - 1.287) |
| Alzheimer’s disease | 1.003 (0.913 - 1.105) | 1.018 (0.908 - 1.127) |
| Vascular dementia | 1.125 (0.822 - 1.424) | 1.020 (0.891 - 1.157) |

CI, confidence interval; HR, hazard ratio; N, number; PSM, propensity score matching. Values from the multivariate competing risk models were adjusted for age, sex, body mass index, household income, smoking status, alcohol consumption, regular physical activity, comorbidities, and Charlson comorbidity index.
