# Supplementary material for: Association between varicose veins and occurrence of dementia: A nationwide population-based cohort study
Source: PLoS One. 2025 Apr 30;20(4):e0322892. doi: 10.1371/journal.pone.0322892 (PMC12043132; doi:10.1371/journal.pone.0322892)
Supplement: S7 Table — (DOCX) [file pone.0322892.s009.docx]

**S7 Table.** Frequency table of procedure code for population who received procedure/treatment for varicose vein.

| Hemorrhoids treatment group, N = 1,990 | | | | | | | | | | | |
| --- | --- | --- | --- | --- | --- | --- | --- | --- | --- | --- | --- |
| Number of treatment = 1 | | Number of treatment = 2 | | Number of treatment = 3 | | Number of treatment = 4 | | Number of treatment = 5 | | Number of treatment = 6 | |
| Procedure code | N (%) | Procedure code | N (%) | Procedure code | N (%) | Procedure code | N (%) | Procedure code | N (%) | Procedure code | N (%) |
| O0261 | 116 (5.8%) | O0215 & O0216 | 28 (1.4%) | O0215 & O0216 & O0217 | 6 (0.3%) | O0261 & O0263 & O0264 & O0265 | 1 (0.1%) | O0263 & O0264 & O0265 & O0266 & O2052 | 1 (0.1%) | O0263 & O0265 & O0266 & O0267 & O0216 & O0217 | 1 (0.1%) |
| O0262 | 64 (3.2%) | O0215 & O0217 | 7 (0.4%) | O0261 & O0215 & O0216 | 1 (0.1%) | O0261 & O0263 & O0264 & O2052 | 1 (0.1%) |  |  |  |  |
| O0263 | 134 (5.7%) | O0216 & O0217 | 25 (1.3%) | O0261 & O0215 & O0217 | 1 (0.1%) | O0261 & O0263 & O0265 & O0216 | 2 (0.1%) |  |  |  |  |
| O0264 | 165 (8.3%) | O0261 & O0215 | 9 (0.5%) | O0261 & O0263 & O2052 | 1 (0.1%) | O0261 & O0264 & O0265 & O2052 | 1 (0.1%) |  |  |  |  |
| O0265 | 244 (11.3%) | O0261 & O0216 | 4 (0.2%) | O0261 & O0265 & O2052 | 1 (0.1%) | O0261 & O0265 & O0215 & O0217 | 1 (0.1%) |  |  |  |  |
| O0266 | 149 (7.5%) | O0261 & O0262 | 5 (0.3%) | O0261 & O2052 & O0215 | 2 (0.1%) | O0262 & O0264 & O0216 & O0217 | 1 (0.1%) |  |  |  |  |
| O0267 | 11 (0.6%) | O0261 & O0263 | 7 (0.4%) | O0262 & O0215 & O0216 | 1 (0.1%) | O0262 & O0264 & O0266 & O2052 | 1 (0.1%) |  |  |  |  |
| O2052 | 276 (12.9%) | O0261 & O0265 | 3 (0.2%) | O0262 & O0215 & O0217 | 1 (0.1%) | O0262 & O2052 & O0215 & O0216 | 1 (0.1%) |  |  |  |  |
| O0215 | 206 (9.5%) | O0261 & O0266 | 1 (0.1%) | O0262 & O0264 & O0265 | 2 (0.1%) | O0263 & O0264 & O0265 & O2052 | 1 (0.1%) |  |  |  |  |
| O0216 | 61 (3.1%) | O0261 & O0267 | 1 (0.1%) | O0262 & O0264 & O0266 | 1 (0.1%) | O0263 & O0265 & O0266 & O0217 | 1 (0.1%) |  |  |  |  |
| O0217 | 59 (3%) | O0261 & O2052 | 11 (0.6%) | O0262 & O0266 & O0217 | 1 (0.1%) | O0264 & O0265 & O2052 & O0217 | 1 (0.1%) |  |  |  |  |
|  |  | O0262 & O0215 | 3 (0.2%) | O0263 & O0215 & O0216 | 1 (0.1%) | O0264 & O0266 & O2052 & O0215 | 1 (0.1%) |  |  |  |  |
|  |  | O0262 & O0216 | 2 (0.1%) | O0263 & O0215 & O0217 | 1 (0.1%) | O0264 & O0266 & O2052 & O0216 | 1 (0.1%) |  |  |  |  |
|  |  | O0262 & O0217 | 2 (0.1%) | O0263 & O0264 & O0265 | 2 (0.1%) | O0265 & O0215 & O0216 & O0217 | 1 (0.1%) |  |  |  |  |
|  |  | O0262 & O0263 | 5 (0.3%) | O0263 & O0265 & O0216 | 1 (0.1%) |  |  |  |  |  |  |
|  |  | O0262 & O0264 | 4 (0.2%) | O0263 & O0265 & O0266 | 1 (0.1%) |  |  |  |  |  |  |
|  |  | O0262 & O0265 | 5 (0.3%) | O0263 & O0266 & O0216 | 1 (0.1%) |  |  |  |  |  |  |
|  |  | O0262 & O0266 | 2 (0.1%) | O0263 & O0266 & O0217 | 3 (0.2%) |  |  |  |  |  |  |
|  |  | O0262 & O2052 | 9 (0.5%) | O0263 & O2052 & O0215 | 1 (0.1%) |  |  |  |  |  |  |
|  |  | O0263 & O0215 | 3 (0.2%) | O0263 & O2052 & O0216 | 1 (0.1%) |  |  |  |  |  |  |
|  |  | O0263 & O0216 | 2 (0.1%) | O0264 & O0215 & O0216 | 3 (0.2%) |  |  |  |  |  |  |
|  |  | O0263 & O0217 | 9 (0.5%) | O0264 & O0216 & O0217 | 1 (0.1%) |  |  |  |  |  |  |
|  |  | O0263 & O0264 | 14 (0.7%) | O0264 & O0265 & O0215 | 2 (0.1%) |  |  |  |  |  |  |
|  |  | O0263 & O0265 | 12 (0.6%) | O0264 & O0265 & O0216 | 2 (0.1%) |  |  |  |  |  |  |
|  |  | O0263 & O0266 | 4 (0.2%) | O0264 & O0265 & O0266 | 3 (0.2%) |  |  |  |  |  |  |
|  |  | O0263 & O0267 | 1 (0.1%) | O0264 & O2052 & O0215 | 2 (0.1%) |  |  |  |  |  |  |
|  |  | O0263 & O2052 | 13 (0.7%) | O0264 & O2052 & O0217 | 1 (0.1%) |  |  |  |  |  |  |
|  |  | O0264 & O0215 | 9 (0.5%) | O0265 & O0215 & O0216 | 3 (0.2%) |  |  |  |  |  |  |
|  |  | O0264 & O0216 | 1 (0.1%) | O0265 & O0215 & O0217 | 1 (0.1%) |  |  |  |  |  |  |
|  |  | O0264 & O0217 | 11 (0.6%) | O0265 & O0216 & O0217 | 1 (0.1%) |  |  |  |  |  |  |
|  |  | O0264 & O0265 | 13 (0.7%) | O0265 & O0266 & O0215 | 1 (0.1%) |  |  |  |  |  |  |
|  |  | O0264 & O0266 | 26 (1.3%) | O0265 & O0266 & O0216 | 2 (0.1%) |  |  |  |  |  |  |
|  |  | O0264 & O2052 | 15 (0.8%) | O0265 & O0266 & O0217 | 3 (0.2%) |  |  |  |  |  |  |
|  |  | O0265 & O0215 | 12 (0.6%) | O0265 & O0266 & O2052 | 3 (0.2%) |  |  |  |  |  |  |
|  |  | O0265 & O0216 | 7 (0.4%) | O0265 & O0267 & O2052 | 1 (0.1%) |  |  |  |  |  |  |
|  |  | O0265 & O0217 | 10 (0.5%) | O0265 & O2052 & O0215 | 2 (0.1%) |  |  |  |  |  |  |
|  |  | O0265 & O0266 | 18 (0.9%) | O0265 & O2052 & O0216 | 1 (0.1%) |  |  |  |  |  |  |
|  |  | O0265 & O0267 | 1 (0.1%) | O0265 & O2052 & O0217 | 1 (0.1%) |  |  |  |  |  |  |
|  |  | O0265 & O2052 | 18 (0.9%) | O0266 & O0215 & O0216 | 1 (0.1%) |  |  |  |  |  |  |
|  |  | O0266 & O0215 | 18 (0.9%) | O0266 & O0215 & O0217 | 1 (0.1%) |  |  |  |  |  |  |
|  |  | O0266 & O0216 | 1 (0.1%) | O0266 & O0216 & O0217 | 1 (0.1%) |  |  |  |  |  |  |
|  |  | O0266 & O0217 | 18 (0.9%) | O0266 & O2052 & O0217 | 1 (0.1%) |  |  |  |  |  |  |
|  |  | O0266 & O2052 | 8 (0.4%) | O2052 & O0215 & O0216 | 3 (0.2%) |  |  |  |  |  |  |
|  |  | O0267 & O0215 | 1 (0.1%) | O2052 & O0216 & O0217 | 3 (0.2%) |  |  |  |  |  |  |
|  |  | O2052 & O0215 | 13 (0.7%) |  |  |  |  |  |  |  |  |
|  |  | O2052 & O0216 | 12 (0.6%) |  |  |  |  |  |  |  |  |
|  |  | O2052 & O0217 | 12 (0.6%) |  |  |  |  |  |  |  |  |

Abbreviations: N, number; The procedure code names of the treatment corresponding to each codes are as follows: Saphenous vein ligation & stab avulsion + perforator ligation(O0261); Saphenous vein ligation & stab avulsion – perforator ligation (O0262); Segmental stripping & stab avulsion + perforator ligation(O0263); Segmental stripping & stab avulsion – perforator ligation(O0264); Total stripping & stab avulsion + perforator ligation(O0265); Total stripping & stab avulsion – perforator ligation(O0266); Varicose vein operation, others(perineum)(O0267); Local resection(O2052); 1–3 sites(O0215); 4–6 sites (O0216); More than 7 sites(O0217).
