# Supplementary material for: Association between varicose veins and occurrence of dementia: A nationwide population-based cohort study
Source: PLoS One. 2025 Apr 30;20(4):e0322892. doi: 10.1371/journal.pone.0322892 (PMC12043132; doi:10.1371/journal.pone.0322892)
Supplement: S8 Table — (DOCX) [file pone.0322892.s010.docx]

**S8 Table.** Results of Cox regression analysis for the association of procedure/treatment for varicose vein with risk of all-cause dementia.

| Variables | Before PSM  N = 5,096 | After PSM 1:1 N = 3,882 |
| --- | --- | --- |
|  | Adjusted  HR (95% CI) | Adjusted  HR (95% CI) |
| Without treatment | Reference | Reference |
| With treatment | 0.884 (0.778 - 1.005) | 0.920 (0.796 - 1.062) |
| Age, years | 1.100 (1.092 - 1.108) | 1.104 (1.094 - 1.114) |
| Sex |  |  |
| Male | Reference | Reference |
| Female | 1.466 (1.256 - 1.711) | 1.542 (1.277 - 1.862) |
| Body mass index (kg/m2) | 0.998 (0.978 - 1.019) | 1.014 (0.989 - 1.040) |
| Household income |  |  |
| Low | Reference | Reference |
| Middle | 0.979 (0.849 - 1.129) | 0.973 (0.822 - 1.153) |
| High | 0.770 (0.662 - 0.896) | 0.708 (0.589 - 0.851) |
| Smoking status |  |  |
| Never | Reference | Reference |
| Former | 0.926 (0.724 - 1.185) | 1.072 (0.807 - 1.425) |
| Current | 0.983 (0.781 - 1.238) | 1.003 (0.747 - 1.346) |
| Alcohol consumption (days/week) |  |  |
| None | Reference | Reference |
| 1 - 2 times | 0.910 (0.768 - 1.078) | 0.891 (0.730 - 1.089) |
| 3 - 4 times | 1.215 (0.917 - 1.609) | 1.013 (0.708 - 1.448) |
| ≥ 5 times | 1.176 (0.849 - 1.628) | 1.345 (0.903 - 2.003) |
| Regular physical activity (days/week) |  |  |
| None | Reference | Reference |
| 1 - 4 days | 0.923 (0.804 - 1.059) | 0.887 (0.750 - 1.048) |
| ≥ 5 days | 0.981 (0.841 - 1.145) | 0.931 (0.772 - 1.122) |
| Comorbidities |  |  |
| Hypertension | 1.059 (0.927 - 1.210) | 1.052 (0.893 - 1.239) |
| Diabetes mellitus | 1.121 (0.928 - 1.354) | 1.100 (0.863 - 1.403) |
| Dyslipidemia | 1.140 (0.997 - 1.303) | 1.126 (0.957 - 1.324) |
| Stroke | 1.529 (0.859 - 2.720) | 1.347 (0.689 - 2.632) |
| Myocardial Infarction | 2.018 (0.950 - 4.288) | 2.545 (0.939 - 6.897) |
| COPD | 1.245 (1.102 - 1.405) | 1.174 (1.013 - 1.361) |
| Renal disease | 1.044 (0.835 - 1.304) | 1.081 (0.805 - 1.451) |
| Liver disease | 1.245 (1.094 - 1.418) | 1.209 (1.030 - 1.419) |
| Cancer | 0.967 (0.784 - 1.192) | 0.943 (0.722 - 1.232) |
| Charlson comorbidity index |  |  |
| 0 | Reference | Reference |
| 1 | 0.919 (0.717 - 1.179) | 0.976 (0.699 - 1.362) |
| ≥ 2 | 0.891 (0.396 - 2.008) | 0.751 (0.239 - 2.362) |

Abbreviations: CI, confidence interval; COPD, chronic obstructive pulmonary disease; HR, hazard ratio; N, number; PSM, propensity score matching.
