# Supplementary material for: Association between varicose veins and occurrence of dementia: A nationwide population-based cohort study
Source: PLoS One. 2025 Apr 30;20(4):e0322892. doi: 10.1371/journal.pone.0322892 (PMC12043132; doi:10.1371/journal.pone.0322892)
Supplement: S9 Table — (DOCX) [file pone.0322892.s011.docx]

**S9 Table.** Results of Cox regression analysis for the association of procedure/treatment for varicose vein with risk of Alzheimer’s disease.

| Variables | Before PSM  N = 5,096 | After PSM 1:1 N = 3,882 |
| --- | --- | --- |
|  | Adjusted  HR (95% CI) | Adjusted  HR (95% CI) |
| Without treatment | Reference | Reference |
| With treatment | 0.778 (0.624 - 0.970) | 0.787 (0.615 - 1.007) |
| Age, years | 1.138 (1.125 - 1.152) | 1.150 (1.132 - 1.168) |
| Sex |  |  |
| Male | Reference | Reference |
| Female | 1.311 (1.024 - 1.679) | 1.391 (1.014 - 1.908) |
| Body mass index (kg/m2) | 0.985 (0.952 - 1.019) | 0.989 (0.947 - 1.033) |
| Household income |  |  |
| Low | Reference | Reference |
| Middle | 0.987 (0.779 - 1.251) | 0.940 (0.703 - 1.256) |
| High | 0.862 (0.674 - 1.102) | 0.754 (0.554 - 1.027) |
| Smoking status |  |  |
| Never | Reference | Reference |
| Former | 1.142 (0.786 - 1.661) | 1.520 (0.986 - 2.344) |
| Current | 0.922 (0.630 - 1.348) | 0.916 (0.551 - 1.522) |
| Alcohol consumption (days/week) |  |  |
| None | Reference | Reference |
| 1 - 2 times | 0.869 (0.653 - 1.156) | 0.839 (0.592 - 1.187) |
| 3 - 4 times | 1.005 (0.626 - 1.616) | 0.975 (0.541 - 1.755) |
| ≥ 5 times | 0.924 (0.548 - 1.556) | 1.291 (0.683 - 2.440) |
| Regular physical activity (days/week) |  |  |
| None | Reference | Reference |
| 1 - 4 days | 0.830 (0.659 - 1.046) | 0.823 (0.616 - 1.100) |
| ≥ 5 days | 0.923 (0.723 - 1.178) | 0.913 (0.672 - 1.240) |
| Comorbidities |  |  |
| Hypertension | 0.996 (0.806 - 1.231) | 0.961 (0.735 - 1.258) |
| Diabetes mellitus | 1.449 (1.100 - 1.910) | 1.316 (0.907 - 1.910) |
| Dyslipidemia | 1.072 (0.860 - 1.335) | 1.177 (0.895 - 1.546) |
| Stroke | 1.566 (0.691 - 3.548) | 1.195 (0.437 - 3.267) |
| Myocardial Infarction | 0.434 (0.060 - 3.126) | <.001 (<.001 - >999.999) |
| COPD | 1.291 (1.059 - 1.573) | 1.334 (1.040 - 1.711) |
| Renal disease | 1.104 (0.790 - 1.544) | 1.045 (0.647 - 1.685) |
| Liver disease | 1.103 (0.893 - 1.364) | 0.951 (0.722 - 1.253) |
| Cancer | 0.705 (0.489 - 1.017) | 0.637 (0.386 - 1.053) |
| Charlson comorbidity index |  |  |
| 0 | Reference | Reference |
| 1 | 0.978 (0.665 - 1.438) | 0.843 (0.468 - 1.518) |
| ≥ 2 | 2.174 (0.881 - 5.365) | 1.630 (0.395 - 6.721) |

Abbreviations: CI, confidence interval; COPD, chronic obstructive pulmonary disease; HR, hazard ratio; N, number; PSM, propensity score matching.
