# Supplementary material for: Association between varicose veins and occurrence of dementia: A nationwide population-based cohort study
Source: PLoS One. 2025 Apr 30;20(4):e0322892. doi: 10.1371/journal.pone.0322892 (PMC12043132; doi:10.1371/journal.pone.0322892)
Supplement: S10 Table — (DOCX) [file pone.0322892.s012.docx]

**S10 Table.** Results of Cox regression analysis for the association of procedure/treatment for varicose vein with risk of vascular dementia.

| Variables | Before PSM  N = 5,096 | After PSM 1:1 N = 3,882 |
| --- | --- | --- |
|  | Adjusted  HR (95% CI) | Adjusted  HR (95% CI) |
| Without treatment | Reference | Reference |
| With treatment | 0.630 (0.437 - 0.908) | 0.566 (0.382 - 0.841) |
| Age, years | 1.106 (1.085 - 1.127) | 1.114 (1.088 - 1.140) |
| Sex |  |  |
| Male | Reference | Reference |
| Female | 1.335 (0.891 - 2.000) | 1.629 (0.975 - 2.723) |
| Body mass index (kg/m2) | 0.989 (0.936 - 1.046) | 1.015 (0.950 - 1.084) |
| Household income |  |  |
| Low | Reference | Reference |
| Middle | 0.653 (0.447 - 0.953) | 0.636 (0.395 - 1.024) |
| High | 0.717 (0.493 - 1.042) | 0.910 (0.585 - 1.413) |
| Smoking status |  |  |
| Never | Reference | Reference |
| Former | 1.402 (0.821 - 2.396) | 2.242 (1.196 - 4.202) |
| Current | 0.938 (0.513 - 1.716) | 1.612 (0.777 - 3.344) |
| Alcohol consumption (days/week) |  |  |
| None | Reference | Reference |
| 1 - 2 times | 1.173 (0.764 - 1.801) | 0.818 (0.481 - 1.389) |
| 3 - 4 times | 1.325 (0.672 - 2.614) | 0.886 (0.360 - 2.185) |
| ≥ 5 times | 1.348 (0.620 - 2.930) | 1.191 (0.474 - 2.991) |
| Regular physical activity (days/week) |  |  |
| None | Reference | Reference |
| 1 - 4 days | 0.952 (0.656 - 1.380) | 1.081 (0.687 - 1.701) |
| ≥ 5 days | 1.125 (0.767 - 1.651) | 1.180 (0.737 - 1.890) |
| Comorbidities |  |  |
| Hypertension | 1.216 (0.872 - 1.695) | 1.496 (1.006 - 2.223) |
| Diabetes mellitus | 1.109 (0.703 - 1.749) | 1.116 (0.623 - 1.997) |
| Dyslipidemia | 1.445 (1.012 - 2.063) | 1.437 (0.935 - 2.209) |
| Stroke | 2.959 (1.188 - 7.368) | 2.404 (0.853 - 6.781) |
| Myocardial Infarction | 1.680 (0.231 - 12.239) | 3.288 (0.435 - 24.849) |
| COPD | 1.037 (0.756 - 1.423) | 1.217 (0.829 - 1.787) |
| Renal disease | 1.439 (0.890 - 2.326) | 1.308 (0.687 - 2.491) |
| Liver disease | 1.217 (0.870 - 1.701) | 1.099 (0.726 - 1.662) |
| Cancer | 0.830 (0.486 - 1.417) | 0.931 (0.488 - 1.775) |
| Charlson comorbidity index |  |  |
| 0 | Reference | Reference |
| 1 | 0.682 (0.333 - 1.396) | 1.143 (0.525 - 2.489) |
| ≥ 2 | 2.862 (0.878 - 9.328) | 3.120 (0.722 - 13.487) |

Abbreviations: CI, confidence interval; COPD, chronic obstructive pulmonary disease; HR, hazard ratio; N, number; PSM, propensity score matching.
