# Supplementary material for: Association between varicose veins and occurrence of dementia: A nationwide population-based cohort study
Source: PLoS One. 2025 Apr 30;20(4):e0322892. doi: 10.1371/journal.pone.0322892 (PMC12043132; doi:10.1371/journal.pone.0322892)
Supplement: S11 Table — (DOCX) [file pone.0322892.s013.docx]

**S11 Table.** Results of Cox regression analysis for the association of procedure/treatment for varicose vein with incidence risk of dementia: A 1 - year landmark analysis.

| Variable | Before PSM  N = 5,065 | | | After PSM 1:1 N = 3,868 | | |
| --- | --- | --- | --- | --- | --- | --- |
|  | Incidence rate  (per 100,000 person - years) | Crude  HR (95% CI) | Adjusted  HR (95% CI) | Incidence rate  (per 100,000 person - years) | Crude  HR (95% CI) | Adjusted  HR (95% CI) |
| All - cause dementia | 2,064.807 | 0.747 (0.657 - 0.848) | 0.895 (0.787 - 1.019) | 1,786.467 | 0.862 (0.727 - 1.021) | 0.928 (0.754 - 1.142) |
| Alzheimer’s disease | 742.604 | 0.559 (0.449 - 0.696) | 0.764 (0.610 - 0.956) | 547.420 | 0.688 (0.514 - 0.919) | 0.683 (0.452 - 1.033) |
| Vascular dementia | 282.334 | 0.512 (0.357 - 0.735) | 0.639 (0.441 - 0.926) | 233.860 | 0.526 (0.338 - 0.819) | 0.222 (0.082 - 0.605) |

Abbreviations: PSM, propensity score matching; N, number; HR, hazard ratio; CI, confidence interval. Values from multivariate Cox regression models adjusted for age, sex, body mass index, household income, smoking status, alcohol consumption, regular physical activity, comorbidities, and Charlson comorbidity index
