# Supplementary figures and images for: Association between varicose veins and occurrence of dementia: A nationwide population-based cohort study
Source: PLoS One. 2025 Apr 30;20(4):e0322892. doi: 10.1371/journal.pone.0322892 (PMC12043132; doi:10.1371/journal.pone.0322892)

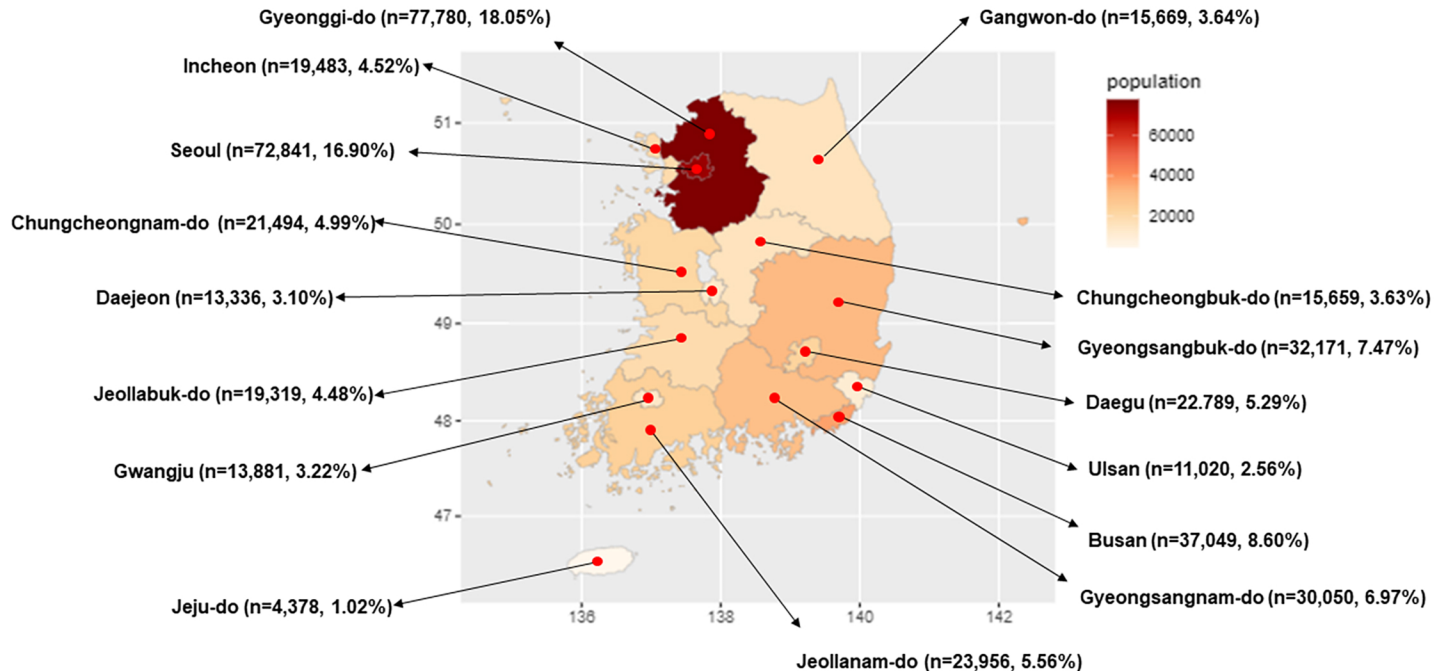

Supplement: S1 Fig — (PDF) [file pone.0322892.s015.pdf]

A) Before matching

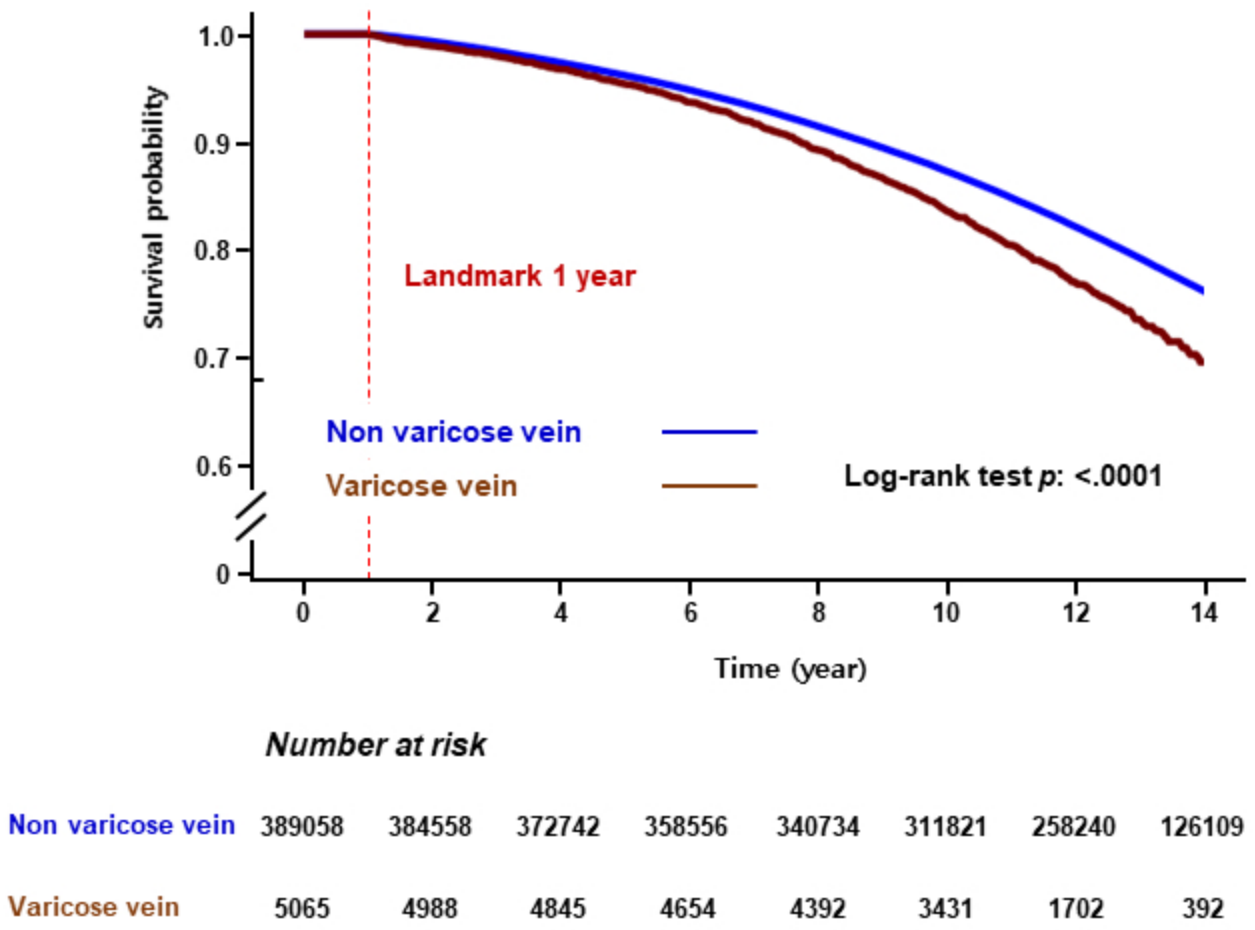

B) After matching

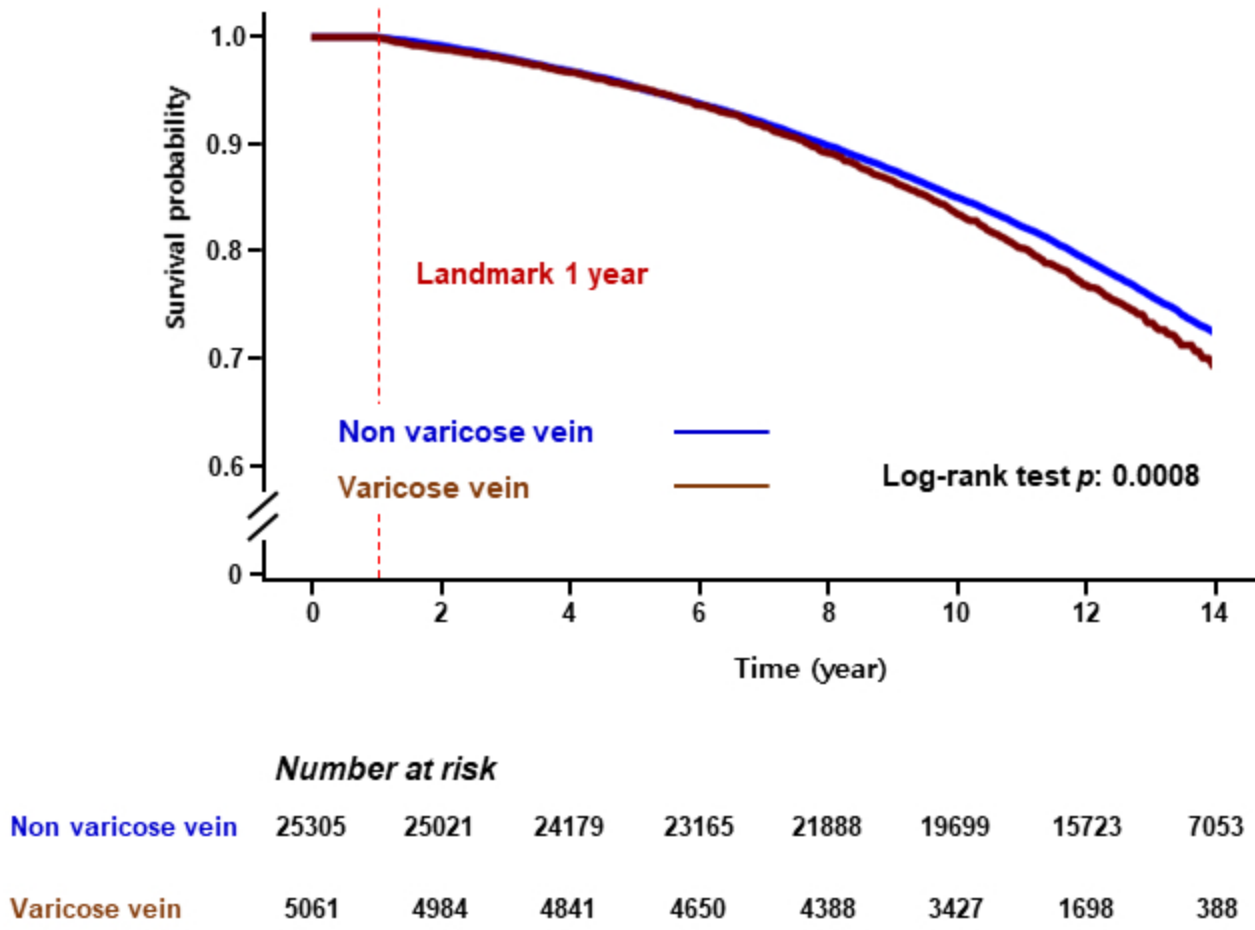

Supplement: S2 Fig — (PDF) [file pone.0322892.s016.pdf]
